# Preeclampsia Susceptibility Assessment Based on Deep Learning Modeling and Single Nucleotide Polymorphism Analysis

**DOI:** 10.3390/biomedicines11051257

**Published:** 2023-04-24

**Authors:** Aida Saadaty, Sara Parhoudeh, Khalil Khashei Varnamkhasti, Mehdi Moghanibashi, Sirous Naeimi

**Affiliations:** 1 Department of Genetics, College of Science, Kazerun Branch, Islamic Azad University, Kazerun 73, Iran; 2 Department of Medical Laboratory Sciences, Faculty of Medicine, Kazerun Branch, Islamic Azad University, Kazerun 73, Iran; 3 Department of Genetics, Faculty of Medicine, Kazerun Branch, Islamic Azad University, Kazerun 73, Iran

**Keywords:** preeclampsia, deep learning modeling, polymorphism, interleukin-13, interleukin-4

## Abstract

The early diagnosis of preeclampsia, a key outlook in improving pregnancy outcomes, still remains elusive. The present study aimed to examine the interleukin-13 and interleukin-4 pathway potential in the early detection of preeclampsia as well as the relationship between interleukin-13 rs2069740(T/A) and rs34255686(C/A) polymorphisms and preeclampsia risk to present a combined model. This study utilized raw data from the GSE149440 microarray dataset, and an expression matrix was constructed using the RMA method and affy package. The genes related to the interleukin-13 and interleukin-4 pathway were extracted from the GSEA, and their expression levels were applied to design multilayer perceptron and PPI graph convolutional neural network models. Moreover, genotyping for the rs2069740(T/A) and rs34255686(C/A) polymorphisms of the interleukin-13 gene were tested using the amplification refractory mutation system PCR method. The outcomes revealed that the expression levels of interleukin-4 and interleukin-13 pathway genes could significantly differentiate early preeclampsia from normal pregnancy. Moreover, the present study’s data suggested significant differences in the genotype distribution, the allelic frequencies and some of the risk markers of the study, in the position of rs34255686 and rs2069740 polymorphisms between the case and control groups. A combined test of two single nucleotide polymorphisms and an expression-based deep learning model could be designed for future preeclampsia diagnostic purposes.

## 1. Introduction

Pregnancy complications associated with new-onset elevated hypertension (systolic >140 mm Hg and diastolic >90 mm Hg on two occasions at least 4 h apart or shorter interval timing of systolic >160 mm Hg and diastolic >110 mm Hg) after 20 weeks’ gestation with accompanying proteinuria (≥300 mg upon 24 h urine collection; or UPCR ≥0.3 mg/mg; or urine protein dipstick reading ≥2+) is defined as preeclampsia. The frequency of other abnormal lab values such as thrombocytopenia, impaired liver function, epigastric pain, pulmonary edema or renal insufficiency can be categorized as severe preeclampsia [1,2]. This important pregnancy pathogenesis complicates 8–10% of all pregnancies and is a major cause of neonatal and maternal morbidity and mortality worldwide [3]. Moreover, this complication elevates the future risk of developing diabetes and cardiovascular disease for women suffering from preeclampsia and both short- and long-term consequences in their offspring [4]. The exact pathogenic molecular mechanisms of preeclampsia are still unclear and, except for delivery, no practical method for resolving preeclampsia symptoms is available [5]. Despite the fact that the etiology of preeclampsia has not yet been fully elucidated, the inflammatory response is known as a hallmark of preeclampsia, so pregnancy itself is a controlled inflammatory condition; however, preeclampsia can be classified as a pro-inflammatory state [6].

It is thought that cytokines as immunological modulators are involved in the development and progression of this pro-inflammatory situation. In normal pregnancy, T helper 2 cytokines (anti-inflammatory) such as interleukins (ILs) 4, 5 and 13 help to neutralize pro-inflammatory cytokines (T helper 1) such as interleukin 2 and gamma interferon. Several studies have noticed the imbalanced concentration of these pro- and anti-inflammatory cytokines in preeclampsia [7,8]. Genetic predisposition is thought to be responsible for the alteration of individual cytokine concentrations or their ratio for the pathophysiology of preeclampsia [9]. Several genetic studies have stated that various gene polymorphisms were associated with the risk of preeclampsia [10]. Cytokine genes’ single nucleotide polymorphisms (SNPs) may affect cytokine transcription and influence its expression level [11]. On the other hand, the role of interleukin-13 (IL-13) and interleukin-4 (IL-4) pathway-related genes, such as STAT1, SOCS1 and JAK, in preeclampsia has been investigated [12]. Although studies suggest the importance of expression alternations and SNPs in cytokines, each of them has its own drawbacks, and a combination of both tests is needed for diagnostic purposes. Therefore, the hypothesis of significant expression changes and mutations in the IL-13 and IL-4 pathway’s key cytokines should be studied.

There are two main methods to evaluate the expression changes in a specific pathway. The first one applies statistical models to see which genes are differentially expressed by comparing normal individuals and patients [13,14]. Then, it finds those up- or downregulated genes included in the pathway and uses them to diagnose the disorder based on the genes found. This method considers each gene separately, which causes low accuracy. However, the second method applies expression patterns of a specific pathway. This method uses gene profiles and their particular expression patterns to diagnose a disease [15]. This study applied the second method and used two deep learning algorithms to diagnose preeclampsia based on IL-4 and IL-13 pathway expression patterns. Two of the leading deep learning structures are the multilayer perceptron (MLP) and graph convolutional neural network (GCNN). This study used both of them for designing its models to select the best one with better metrics. Moreover, two additional specific SNPs in IL-13 were tested to cover the expression of deep learning model defects. Therefore, the present study aimed to study the relationship between preeclampsia risk and rs2069740 (T to A exchange) and rs34255686 (C to A exchange) polymorphisms of IL-13 while evaluating the expression patterns of IL-13 and IL-4 pathway genes in this disease.

## 2. Methods

### 2.1. Data Collection and Preprocessing

In the present study, we first searched the terms “Preeclampsia” and “Normal Pregnancy” in the Gene Expression Omnibus (GEO, https://www.ncbi.nlm.nih.gov/geo/) database on 3 May 2022. The database contains a vast number of datasets related to various studies. We selected the GSE149440 microarray dataset which combined the GSE149434, GSE149436 and GSE149437 datasets. The dataset contained 735 whole blood samples and three different groups (“Normal Pregnancy”, “Early Preeclampsia” and “Preterm Birth”). The platform used was GPL28460 or Affymetrix Human Transcriptome Array 2.0, including 32,800 probes. First, raw CEL files were downloaded through the GEOquery package in R. Initial preprocessing was performed on the data, including backlight removal, data normalization based on the RMA method and data transfer to a logarithmic basis based on 2 using the affy package. Samples with the preterm birth condition were excluded from the study, and only early preeclampsia (<34 weeks because the rate of morbidities declines considerably after this gestational age in women with preeclampsia and the induction of labor is recommended after 34 weeks of gestation) and normal pregnancy samples remained. As the dataset contained multiple studies, batch effect removal was conducted using the limma package.

### 2.2. Deep Learning Neural Network Model Design

We applied neural networks and deep learning to classify healthy and preeclampsia blood samples. There are many types of neural networks; here, we selected MLP and CNN. To design the CNN, a dataset in the form of images was needed. To convert the format of our dataset into an image format, we applied the method used in [16]. First, we downloaded the human protein–protein interaction network from the Stanford Network Analysis Project (SNAP, https://snap.stanford.edu/) on 10 May 2022. It is a CSV file containing one column that shows protein–protein interactions organized by their Entrez ID, each one separated by a comma. For instance, the interaction between GAPDH and GRM1 is shown by 2597, 2911. Then, a graph of the interactions was constructed, each node representing a gene and each vertex representing its expression. The edges show the connections between the proteins and the graph type is an undirected graph because the impact of each protein on the other one is unknown based on the collected data. The graph structure at first was designed for all protein interactions in GSE149440. Then, the IL-13 and IL-4 signaling pathway gene list was downloaded from the GSEA (https://www.gsea-msigdb.org/) on 20 May 2022, which linked us to the Reactome database (https://reactome.org/). Afterwards, all interactions except those between the mentioned pathway genes were excluded from the constructed graph. To be able to pool graph signals, we first needed to coarsen the graph (i.e., to find out which vertices to group together). Then, we ended up having multiple graphs—not unlike a pyramid—with each at one level of resolution. The finest graph is where the input data lies. The coarsest graph is where the data at the output of the graph convolutional layers lies. Then, we computed the Laplacian graph for each of our graphs, defined by their adjacency matrices. For learning, we used a CNN graph with the following hyperparameters. Two convolutional layers were employed with 32 convolutional filters and a polynomial order of 20. Size 2 maximum pooling was applied to both convolutional layers. Two fully connected layers, on the other hand, had 512 and 128 units, respectively. The activation function of a rectified linear unit (ReLU) was used and cross-entropy loss was kept to a minimum. For MLP, four layers were constructed, with 111, 42, 21 and 10 ReLUs, respectively. In MLP, only the expression matrix of the pathway genes was considered as the input, which contained 111 genes as input features.

### 2.3. Experimental Design

For the case-control study, approved by the Islamic Azad University Kazerun Branch Ethics Committee (IR.IAU.KAU.REC.1398.004), conducted in 2019 and 2020, 150 preeclampsia patients and 150 normal healthy pregnant women from Zeynabieh Hospital in Shiraz, Iran, were selected. Demographic characteristics are shown in Table 1. Before entering the study groups, written informed consent was gained from all the participants. Preeclamptic women were included who presented the signs and symptoms of systole >140 mm Hg and diastole >90 mm Hg on two occasions at least 4 h apart (the mild form of preeclampsia) or a shorter interval timing of systole >160 mm Hg and diastole >110 mm Hg (the severe form of preeclampsia) after 20 weeks’ gestation with accompanying proteinuria ≥300 mg upon a 24 h urine collection; or UPCR ≥ 0.3 mg/mg; or a urine protein dipstick reading ≥2+. The control group was healthy pregnant women, without hypertension, with the same gestational age (≥20 weeks), who had been referred to the midwifery clinic of the same hospital to receive prenatal care within the same timeframe. The exclusion criteria were defined as pregnant women with a history of chronic hypertension and current antihypertensive treatment, autoimmune and any cardiac/renal disease, and liver disease/diabetic neuropathy.

### 2.4. DNA Extraction and Genotyping of Polymorphisms

After collecting 5 mL peripheral blood samples in EDTA tubes, genomic DNA was extracted using a Genomic DNA Isolation Kit (GeNet Bio, Daejeon, Korea), using the salting out method. To genotype rs2069740(T/A) and rs34255686(C/A) polymorphisms, the amplification refractory mutation system PCR (ARMS-PCR) method was carried out. For each polymorphism, two external primers (to check the performance of the PCR) and two allele-specific internal primers (to detect each allele) were designed (the designed primer sequences are reported in Table 2). PCR was performed in a total volume of 23.1 μL, including 1 μL of template DNA, 11 μL of 2× Master Mix RED (Ampliqon), 0.8 μL (0.39 pmol) of each primer and 7.9 μL of H_2_O. The cycling conditions were as follows: initial denaturation for 5 min at 94 °C, followed by 30 cycles at 94 °C for 40 s (denaturation), annealing temperature as in Table 2 for 40 s, 1 min at 72 °C (extension) and a final extension for 7 min at 72 °C. PCR products were run by standard electrophoresis on 2% agarose gel for 10 min and visualized on a UV transilluminator. The band lengths are presented in Table 2.

### 2.5. Software and Statistics

All data preprocessing and RMA calculations were conducted in R version 4.1.0. The deep learning model design was conducted in Python 3.10 and using the TensorFlow package. The figures were drawn in GraphPad Prism 9. On the other hand, all samples collected were analyzed using SPSS 19.0 (SPSS Inc., Chicago, IL, USA). Chi-square testing was performed to evaluate the differences in the allele and genotype frequencies. *P* values less than 0.05 were considered statistically significant.

## 3. Results

### 3.1. 111 Genes Are Connected to the IL-13 and IL-4 Signalling Pathway

Using the GSE149440, an expression matrix was constructed based on the RMA method. As this study only needed early preeclampsia and normal blood samples, 355 out of the 735 samples were removed from the matrix. The number of samples is demonstrated in Table 3. A total of 380 samples remained, 66 of which were of early preeclampsia and 314 of the control. IL-4 and IL-13 pathway gene expression levels were extracted from the modified expression matrix. The genes linked to this pathway are shown in Supplementary Table S1. Moreover, the expression matrix contained 32 batches, so we removed their effect through the limma package and prepared a new expression matrix for designing deep learning models, as shown in Supplementary Table S2. However, because the studies used the same platform and methods for data generation, the batch effect could not affect the data considerably.

### 3.2. Two Deep Learning Models to Diagnose Preeclampsia through IL-13 and IL-4 Pathway Expression Data

**Figure 1:**
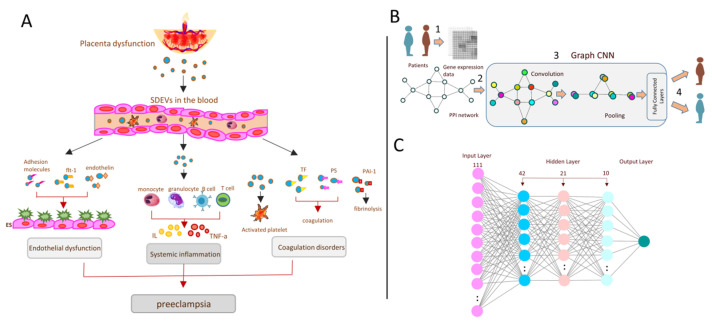
Prediction of preeclampsia from systemic inflammation through deep learning methods. (**A**) The main dysfunctions and disorders related to preeclampsia. As shown, SDEV release in the blood through placental dysfunction might affect endothelial cells, immune cells and the coagulation process, which might lead to preeclampsia disorder. In this study, only one of the pathways related to systemic inflammation was studied. (**B**) Graph of the convolutional neural network (GCNN) summary. Microarray expression data were taken from patients, and normal samples were used to build the IL-4 and IL-13 pathway graph. Then, convolutional and pooling layers were applied for classification. (**C**) The structure of the multilayer perceptron (MLP) model. The model contains three hidden layers with ReLUs as the activation functions for all of them. The input layer also contains 111 units representing all 111 genes linked to the mentioned pathway.

In the present study, we designed two deep learning models for diagnostic purposes, GCNN and MLP. The GCNN model used a structure based on IL-13 and IL-4 pathway connections. This means that the model considered the biological aspects of the hypothesis. On the other hand, the MLP model only used the expression matrix as given features for model training. Therefore, we aimed to compare the accuracy of the pathway connection-based model with the usual MLP model that did not consider any biological background. The structure of each model is depicted in Figure 1A–C. Although The outcomes of both models showed high accuracy (Table 4), the MLP model classification power was better than the GCNN model. Although both models could significantly differentiate normal pregnancy from preeclampsia, there was still about a 20% error in them that needed to be resolved.

### 3.3. The Significant SNPs in IL-13 the Gene Might Improve the Accuracy of the Expression-Based Deep Learning Model

According to the genotype and allele frequencies of the IL-13 rs2069740(T/A) and rs34255686(C/A) polymorphisms summarized in Table 5, the frequency of alleles in both polymorphic positions (*p*_rs2069740_ = 0.009; *p*_rs34255686_ = 0.00) and the genotype frequencies for rs34255686(C/A) polymorphisms (*p* = 0.002) differed significantly between the patient and control groups. No significant difference was observed between the studied groups in the genotype frequency of rs2069740(T/A) polymorphisms (*p* = 0.099). Moreover, there was no significant difference in the combined genotype analyses except for the TT–AA genotypes (*p* = 0.001).

Further, we analyzed the association between preeclampsia risk markers such as seizures, proteinuria, edema, diabetes, multipara, hypertension, gravida, hyperthyroidism, HELLP syndrome, history of abortion, and history of preeclampsia and rs2069740(T/A) and rs34255686(C/A) polymorphisms. Except in the cases of HELLP syndrome (*p* = 0.008) and edema (*p* = 0.047) in the position of rs34255686 and multipara (*p* = 0.02) and gravida (*p* = 0.015) markers in the case of rs2069740, no significant difference was detected for other markers (Table 6).

## 4. Discussion

Preeclampsia is a frequent pregnancy condition that is linked to increased maternal morbidity and death, as well as intrauterine fetal development restriction [17]. As the diagnosis of this fatal disorder can be very time-consuming and inaccurate, we aimed to examine a deep learning-based expression model and introduce significant SNPs in preeclampsia for future diagnostic purposes [18].

Deep learning models in preeclampsia diagnosis have been used in some previous research. Previous studies have considered some phenotypic parameters as features for their deep learning model training for preeclampsia. For instance, M Tahir et al. applied 17 phenotypic parameters of women to detect preeclampsia based on some metabolic and physiological features [19]. Another study used long short-term memory (LSTM) neural networks to diagnose preeclampsia based on time series data [20]. Moreover, Q Wang et al. used ultrasonic image features in the diagnosis of perinatal outcomes of severe preeclampsia through a convolutional neural network model [21]. In addition, R Bennett et al. applied an imbalance-aware deep neural network to rank the most critical risk factors of preeclampsia and use them to diagnose it at its early stages [22]. Although all of the previous studies have their benefits, as clinical technologies are improving and the focus of most of them are on a high throughput and molecular data for their high accuracy and detail, we believe that models should move away from this type of data for better prediction and classification. Thus, in this study, we tried for the first time to design a model for the early diagnosis of preeclampsia based on molecular data, such as expression values and SNPs.

Many studies have referred to some genes whose expression or mutations are vital in the occurrence of preeclampsia. We first gathered those genes and found out that many of them are related to cytokines and their related pathways. Then, we selected one of the pathways that was linked to many of the referred genes in previous studies. However, almost none of them directly mentioned the IL-13 and IL-4 signaling pathway; they suggested the genes connected to this pathway as being preeclampsia risk factors, initiators or progressors [23,24,25,26,27,28,29,30,31,32]. Therefore, in the current study, we examined the role of the pathway’s genes in preeclampsia. We designed two models to assess each one’s performance. Obviously, the MLP model revealed better results with an overall accuracy of 82.11% and an average F1-score of 82.32% compared to the GCNN model with an 83.16% overall accuracy and 75.51 weighted average F1-score. However, we suggest that increasing the number of samples could improve the GCNN model results. Moreover, it is possible to consider all gene interactions in the design of the GCNN model. This study did not consider all genes because of the enormous number of features (about 32,000) present against the small number of samples. Moreover, as the connections among genes are limited in one pathway, the GCNN model could not achieve the best performance. Furthermore, as this is the first study that used expression data for deep learning model design, we added a simple SNP test for a better combined test to improve our model. We aimed for a test that could easily be applied in clinics.

Since the role of IL-13 polymorphisms as peptide cytokines belonging to the T helper 2 family and coded by the gene IL-13 in the chromosomal location of 5q31.1 in preeclampsia was not yet revealed by experimental investigation [33], this study aimed to investigate the relationship between IL-13 gene polymorphisms and preeclampsia risk. We selected two possible polymorphic sites of IL-13, including rs2069740(T/A) and rs34255686(C/A). In our study, significant differences were found in the frequency of alleles in both mentioned polymorphic positions and genotype frequencies for rs34255686(C/A) polymorphism between the patient and control groups, but on the contrary, we did not observe differences in the genotype frequencies of rs2069740(T/A) polymorphisms. Moreover, in our study, a significant difference was only observed in the TT–AA combined genotypes between the patient and control groups. So, due to these observed significant differences, rs2069740(T/A) and rs34255686(C/A) polymorphisms of IL-13 were considered relevant with an increased risk of preeclampsia. Moreover, in a similar study, Liu et al. suggested that IL-27 polymorphisms may be involved in the development of preeclampsia in the Chinese population [34]. In a study by Fan et al., the IL-10-592A/C genetic variant was observed to be associated with preeclampsia risk in pregnant women [35]. Song et al. indicated that the IL-10-592A/C (rs1800872) polymorphism was associated with an increased risk of early-onset preeclampsia in a Chinese population [11]. Raguema et al. suggested that the IL-10-819T/T variant and the ATA haplotype, which are associated with the low production of IL-10, represent genetic risk factors for preeclampsia in Tunisian women [36]. Quach et al. showed that an SNP combination (+3027C/C and +3187G/G) was significantly more prevalent in preeclampsia cases [37]. Pacheco et al. also found that 509CT/869TC combined genotypes were significantly associated with an increased risk of preeclampsia [38]. Subsequently, determining the effects of rs2069740(T/A) and rs34255686(C/A) polymorphisms on preeclampsia risk markers showed no significant difference between the two groups for other risk markers, except for HELLP syndrome and edema in the position of rs34255686 and multipara and gravida markers in the position of rs2069740 polymorphisms. PFAB, et al. also reported that carrying the C allele of the interleukin-6 polymorphisms (interleukin-6 S174G > C) was significantly associated with new-onset edema in pregnant women [39]. Wang et al. showed a significant association between the TNF-α-308G/A polymorphism and parity (Wang et al., 2018). Similarly, no significant relation in a study by Lisi et al. was found between preeclampsia risk factors, such as systolic and diastolic blood pressure, proteinuria and endothelin-1 type-A receptor gene polymorphism (7231 G4A) [40]. Although the current report revealed the genetic association between rs2069740(T/A) and rs34255686(C/A) polymorphisms in the IL-13 gene and the risk of preeclampsia, a more significant sample size is required to validate the observations of the present study.

## 5. Conclusions

IL-4 and IL-13 pathways can considerably affect preeclampsia and deep learning models based on these pathway genes predicting patients’ status with high accuracy. We also concluded that polymorphisms of IL-13 in the positions of rs2069740(T/A) and rs34255686(C/A) contribute to the risk of preeclampsia. We believe that this combined test of two single nucleotide polymorphisms and an expression-based deep learning model is able to predict preeclampsia in very early pregnancy. It is worth noting that this model and SNP-based testing could be invaluable in screening high-risk pregnancies in clinical practice and could serve as a decision-making reference for clinicians.

Therefore, we suggest more investigation of a diagnostic model based on both expression and polymorphism results. Finally, to better understand the pathology of the disease due to its life-threatening properties and likely involvement of more polymorphic sites in its pathogenesis, research into more polymorphisms and the expression patterns of IL-4 and IL-13 pathway genes is needed.

## Figures and Tables

**Table 1:** Demographic characteristics of participants in the two groups.

Variable	Preeclampsia	Control	*p*-Value
	*N* = (150)	*N* = (150)	
Age, years	30.09 ± 6.87	27.23 ± 4.86	0.239
Range	16–48	16–38	–
BMI, kg/m^2^	26.19 ± 3.60	25.83 ± 3.47	0.214
Gestational age, median (range), wk (at the time of sampling)	32 (28–33)	32 (28–33)	–
Mode of delivery (at the time of sampling) Emergency caesarean section Pre-labour caesarean section	7 (4.67) 3 (2)	–	–
Systolic blood pressure, mmHg	151.84 ± 10.78	109.37 ± 11.07	<0.001
Diastolic blood pressure, mmHg	94.95 ± 6.88	64.85 ± 9.02	<0.001

**Table 2:** Designed primer sequences for the ARMS-PCR reactions, annealing temperature and amplicon lengths.

SNP	Primer Sequence	Annealing Temperature (°C)	Fragment Length
rs2069740(T/A)	FO:CCTCTGCACAGTTTGGAC RO:TCTGTCCAGCAATCCAGG FI:AATGCCGTGGCCTCTGCT RI:CAGCCTTAGTCCAGGTCAGAGA	58.2 °C	T:151 bp A:384 bp
rs34255686(C/A)	FO: CCTCTGCACAGTTTGGAC RO: TCTGTCCAGCAATCCAGG FI: CCTTCTCAATAAGTCCAT RI: CTGGTTCTGGGTGATGTTT	56.8 °C	C:186 bp A:220 bp

**Table 3:** The grouping information of patients of the GSE140449.

Group	Subgroup	Total Number
Control	TNL(176)	314
	TL(138)	
Early Preeclampsia	None	66
Preterm Birth	None	355

**Table 4:** Model Evaluation.

Model	Accuracy	F1-Score (Weighted)
Graph Convolutional Neural Network (GCNN)	83.16%	75.51%
Multalayer Perceptron (MLP)	82.11%	82.32%

**Table 5:** Genotype and allele frequencies and combined genotypes of the IL-13 (rs2069740(T/A) and rs34255686(C/A)) polymorphisms in preeclamptic and normal pregnant women.

Genotype and Allele	Control Group	Patient Group	*p*-Value
	n (%)	n (%)	
rs2069740(T/A)			
AA	20(13.3)	9(6)	0.099
TT	123(82)	133(88.7)	
AT	7(4.7)	8(5.3)	
A	47(15.7)	26(8.7)	0.009
T	253(84.3)	274(91.3)	
rs34255686(C/A)			
CC	121(80.7)	141(94)	0.002
AA	15(10)	3(2)	
CA	14(9.3)	6(4)	
A	12(4)	44(14.7)	0.00
C	288(96)	256(85.3)	
Genotype combination (rs2069740–rs34255686)			
AA–CC	18(12)	9(6)	0.06
AA–AA	2(1.3)	0(0)	0.1
TT–CC	117(78)	106(70.7)	0.14
TT–AA	0(0)	13(8.7)	0.001
TT–CA	6(4)	14(9.3)	0.06
AT–CC	6(4)	7(4.7)	0.7
AT–AA	1(7)	1(7)	1

**Table 6:** Associations between the IL-13 (rs2069740(T/A) and rs34255686(C/A)) polymorphisms and preeclampsia risk markers in preeclamptic and healthy pregnant women.

rs2069740 (T/A)	Genotype n (%)			*p*-Value	rs34255686 (C/A)	Genotype n (%)			*p*-Value
	AA	TT	AT			CC	AA	CA	
Seizures Present	0 (0)	1 (100)	0 (0)	0.9	Seizures Present	1 (100)	0 (0)	0 (0)	0.88
Absent	9 (6)	132 (88)	8 (5.4)		Absent	120 (80.5)	15 (10)	14 (9.4)	
Proteinuria Absent	3 (5.7)	47 (88.7)	3 (5.7)	0.9	Proteinuria Absent	43 (81)	4 (7)	6 (11)	0.5
Trace	1 (5.6)	16 (88)	1 (5.6)		Trace	16 (88.9)	0 (0)	2 (11)	
1	3 (6.5)	41 (89.3)	2 (4.3)		1	35 (76)	7 (15)	4 (8.7)	
2	2 (13)	12 (80)	1 (6.7)		2	11 (73)	3 (20)	1 (6.7)	
3	0 (0)	13 (92)	1 (7.1)		3	13 (93.1)	0 (0)	1 (6.7)	
4	0 (0)	4 (100)	0 (0)		4	3 (75)	1 (25)	0 (0)	
Edema Absent	0 (0)	23 (100)	0 (0)	0.131	Edema Absent	15 (65)	4 (17)	4 (17)	0.047
1	8 (8.8)	78 (85)	5 (5.5)		1	79 (86)	4 (4.4)	8 (8.8)	
2	0 (0)	24 (96)	1 (4)		2	18 (72)	6 (24)	1 (4)	
3	1 (9.1)	8 (72.7)	2 (18.2)		3	9 (81)	1 (9.1)	1 (9.1)	
Diabetes Present	1 (11)	8 (88)	0 (0)	0.629	Diabetes Present	7 (77.8)	2 (22.2)	0 (0)	0.311
Absent	8 (5.7)	125 (88)	8 (5.7)		Absent	114 (80.7)	13 (9.2)	14 (9.9)	
Multipara Primary	11 (8)	115 (83)	12 (8.7)	0.02	Multipara Primary	122 (88.4)	10 (7.2)	6 (4.3)	0.252
Multiple	18 (11.1)	141 (87)	3 (1.9)		Multiple	140 (86.6)	8 (4.9)	14 (8.6)	
Hypertension Mild	8 (6.9)	101 (87.7)	7 (6.4)	0.7	Hypertension Mild	93 (80)	10 (8.6)	13 (11)	0.38
Moderate	1 (0)	24 (33.3)	1 (17.1)		Moderate	22 (84.6)	3 (11)	1 (3.8)	
Severe	0 (0)	8 (100)	0 (0)		Severe	6 (75)	2 (25)	0 (0)	
Gravida Present	2 (33.3)	4 (66.7)	0 (0)	0.015	Gravida Present	5 (83.3)	1 (16.7)	0 (0)	0.64
Absent	7 (4.9)	129 (89.6)	8 (5.6)		Absent	116 (80.6)	14 (9.7)	14 (9.7)	
Hyperthyroidism Present	0 (0)	16 (94)	1 (5.9)	0.54	Hyperthyroidism Present	14 (82.4)	2 (11.8)	1 (5.9)	0.85
Absent	9 (6.8)	117 (80)	7 (5.3)		Absent	107 (80.1)	13 (9.8)	13 (9.8)	
HELLP syndrome Present	0 (0)	1 (100)	0 (0)	0.9	HELLP syndrome Present	0 (0)	0 (0)	1 (100)	0.008
Absent	9 (6)	132 (88.6)	8 (5.4)		Absent	121 (81.2)	15 (10)	13 (8.7)	
History of abortion Present	1 (3.3)	26 (86)	3 (10)	0.36	History of abortion Present	26 (86.7)	4 (13.3)	0 (0)	0.13
Absent	8 (6.7)	107 (89.2)	5 (4.2)		Absent	95 (79.3)	11 (9.2)	14 (11.7)	
History of preeclampsia Present	1 (7.7)	10 (76.9)	2 (15)	0.225	History of preeclampsia Present	11 (84)	1 (7.7)	1 (7.7)	0.9
Absent	8 (5.8)	123 (89)	6 (4.4)		Absent	110 (80.5)	14 (10)	13 (9.5)	

## Data Availability

The data that support the findings of this study are available from the corresponding author upon reasonable request.

## Supplementary Materials

The following supporting information can be downloaded at https://www.mdpi.com/article/10.3390/biomedicines11051257/s1.

## Abbreviations

SNP	Single nucleotide polymorphism
IL	Interleukin
MLP	Multilayer perceptron
GCNN	Graph convolutional neural network
GEO	Gene Expression Omnibus
SNAP	Stanford Network Analysis Project
ARMS-PCR	Amplification refractory mutation system PCR
