# Supplementary material for: Preeclampsia Susceptibility Assessment Based on Deep Learning Modeling and Single Nucleotide Polymorphism Analysis
Source: Biomedicines. 2023 Apr 24;11(5):1257. doi: 10.3390/biomedicines11051257 (PMC10215843; doi:10.3390/biomedicines11051257)
Supplement: Supplementary file 1 [file biomedicines-11-01257-s001.zip › Supplementry Table SI.pdf]

Supplementary Table SI. The genes related to IL-13 and IL-4 pathway extracted from Reactome Database

Symbol	*Entrez*	*Annot*
IL23R	149233	interleukin 23 receptor(IL23R)
RORC	6097	RAR related orphan receptor C(RORC)
F13A1	2162	coagulation factor XIII A chain(F13A1)
RORA	6095	RAR related orphan receptor A(RORA)
FGF2	2247	fibroblast growth factor 2(FGF2)
TNF	7124	tumour necrosis factor(TNF)
ICAM1	3383	intercellular adhesion molecule 1(ICAM1)
SOX2	6657	SRY-box transcription factor 2(SOX2)
IGHG4	3503	immunoglobulin heavy constant gamma 4 (G4m marker)(IGHG4)
IGHG1	3500	immunoglobulin heavy constant gamma 1 (G1m marker)(IGHG1)
CCND1	595	cyclin D1(CCND1)
MYC	4609	MYC proto-oncogene, bHLH transcription factor(MYC)
PIM1	5292	Pim-1 proto-oncogene, serine/threonine kinase(PIM1)
IL12B	3593	interleukin 12B(IL12B)
AKT1	207	AKT serine/threonine kinase 1(AKT1)
IL12A	3592	interleukin 12A(IL12A)
IL13RA2	3598	interleukin 13 receptor subunit alpha 2(IL13RA2)
JUNB	3726	JunB proto-oncogene, AP-1 transcription factor subunit(JUNB)
IL6R	3570	interleukin 6 receptor(IL6R)
IL13RA1	3597	interleukin 13 receptor subunit alpha 1(IL13RA1)
IL4R	3566	interleukin 4 receptor(IL4R)
HGF	3082	hepatocyte growth factor(HGF)
IL23A	51561	interleukin 23 subunit alpha(IL23A)
FSCN1	6624	fascin actin-bundling protein 1(FSCN1)
TP53	7157	tumor protein p53(TP53)
MAOA	4128	monoamine oxidase A(MAOA)
ALOX15	246	arachidonate 15-lipoxygenase(ALOX15)
TWIST1	7291	twist family bHLH transcription factor 1(TWIST1)
GATA3	2625	GATA binding protein 3(GATA3)
PIK3R1	5295	phosphoinositide-3-kinase regulatory subunit 1(PIK3R1)
FOXO3	2309	forkhead box O3(FOXO3)
HIF1A	3091	hypoxia inducible factor 1 subunit alpha(HIF1A)
FOXO1	2308	forkhead box O1(FOXO1)
HSP90B1	7184	heat shock protein 90 beta family member 1(HSP90B1)
SOCS3	9021	suppressor of cytokine signaling 3(SOCS3)
SOCS1	8651	suppressor of cytokine signaling 1(SOCS1)
NDN	4692	necdin, MAGE family member(NDN)

HMOX1	3162	heme oxygenase 1(HMOX1)
MCL1	4170	MCL1 apoptosis regulator, BCL2 family member(MCL1)
SOCS5	9655	suppressor of cytokine signaling 5(SOCS5)
HSPA8	3312	heat shock protein family A (Hsp70) member 8(HSPA8)
POU2F1	5451	POU class 2 homeobox 1(POU2F1)
TGFB1	7040	transforming growth factor beta 1(TGFB1)
VCAM1	7412	vascular cell adhesion molecule 1(VCAM1)
FN1	2335	fibronectin 1(FN1)
LIF	3976	LIF interleukin 6 family cytokine(LIF)
IL4	3565	interleukin 4(IL4)
IL6	3569	interleukin 6(IL6)
COL1A2	1278	collagen type I alpha 2 chain(COL1A2)
BCL6	604	BCL6 transcription repressor(BCL6)
BCL2	596	BCL2 apoptosis regulator(BCL2)
LCN2	3934	lipocalin 2(LCN2)
NANOG	79923	Nanog homeobox(NANOG)
BCL2L1	598	BCL2 like 1(BCL2L1)
ITGB1	3688	integrin subunit beta 1(ITGB1)
CDKN1A	1026	cyclin dependent kinase inhibitor 1A(CDKN1A)
ITGAM	3684	integrin subunit alpha M(ITGAM)
CXCL8	3576	C-X-C motif chemokine ligand 8(CXCL8)
ITGB2	3689	integrin subunit beta 2(ITGB2)
FASLG	356	Fas ligand(FASLG)
ITGAX	3687	integrin subunit alpha X(ITGAX)
LBP	3929	lipopolysaccharide binding protein(LBP)
TIMP1	7076	TIMP metallopeptidase inhibitor 1(TIMP1)
CD36	948	CD36 molecule(CD36)
JAK2	3717	Janus kinase 2(JAK2)
JAK3	3718	Janus kinase 3(JAK3)
JAK1	3716	Janus kinase 1(JAK1)
IL10	3586	interleukin 10(IL10)
HSP90AA1	3320	heat shock protein 90 alpha family class A member 1(HSP90AA1)
ANXA1	301	annexin A1(ANXA1)
MMP1	4312	matrix metallopeptidase 1(MMP1)
MMP2	4313	matrix metallopeptidase 2(MMP2)
IL13	3596	interleukin 13(IL13)
MMP3	4314	matrix metallopeptidase 3(MMP3)
IL18	3606	interleukin 18(IL18)
FOS	2353	Fos proto-oncogene, AP-1 transcription factor subunit(FOS)
OPRM1	4988	opioid receptor mu 1(OPRM1)
TYK2	7297	tyrosine kinase 2(TYK2)
TNFRSF1B	7133	TNF receptor superfamily member 1B(TNFRSF1B)
MMP9	4318	matrix metallopeptidase 9(MMP9)

FCER2	2208	Fc epsilon receptor II(FCER2)
IL1A	3552	interleukin 1 alpha(IL1A)
ZEB1	6935	zinc finger E-box binding homeobox 1(ZEB1)
IRF4	3662	interferon regulatory factor 4(IRF4)
IL1B	3553	interleukin 1 beta(IL1B)
BIRC5	332	baculoviral IAP repeat containing 5(BIRC5)
IGHE	3497	immunoglobulin heavy constant epsilon(IGHE)
RHOU	58480	ras homolog family member U(RHOU)
OPRD1	4985	opioid receptor delta 1(OPRD1)
LAMA5	3911	laminin subunit alpha 5(LAMA5)
CCL11	6356	C-C motif chemokine ligand 11(CCL11)
CEBPD	1052	CCAAT enhancer binding protein delta(CEBPD)
PTGS2	5743	prostaglandin-endoperoxide synthase 2(PTGS2)
IL2RG	3561	interleukin 2 receptor subunit gamma(IL2RG)
MUC1	4582	mucin 1, cell surface associated(MUC1)
ALOX5	240	arachidonate 5-lipoxygenase(ALOX5)
S1PR1	1901	sphingosine-1-phosphate receptor 1(S1PR1)
CCL2	6347	C-C motif chemokine ligand 2(CCL2)
STAT6	6778	signal transducer and activator of transcription 6(STAT6)
CCL22	6367	C-C motif chemokine ligand 22(CCL22)
NOS2	4843	nitric oxide synthase 2(NOS2)
STAT1	6772	signal transducer and activator of transcription 1(STAT1)
STAT3	6774	signal transducer and activator of transcription 3(STAT3)
OSM	5008	oncostatin M(OSM)
BATF	10538	basic leucine zipper ATF-like transcription factor(BATF)
VEGFA	7422	vascular endothelial growth factor A(VEGFA)
POMC	5443	proopiomelanocortin(POMC)
SAA1	6288	serum amyloid A1(SAA1)
IL17F	112744	interleukin 17F(IL17F)
VIM	7431	vimentin(VIM)
IL17A	3605	interleukin 17A(IL17A)
